# The Relationship between the Transforming Growth Factor *β*1 T29C Gene Polymorphism and Left Ventricular Geometry and Function in Hypertensive Subjects

**DOI:** 10.4061/2010/647147

**Published:** 2010-03-23

**Authors:** Rosario Scaglione, Christiano Argano, Giovanni Duro, Tiziana Di Chiara, Domenico Nuzzo, Daniela Colomba, Maria Cristina Fiore, Salvatore Corrao, Giuseppe Licata

**Affiliations:** ^1^Dipartimento BioMedico di Medicina Interna e Specialistica, University of Palermo, 90144 Palermo, Italy; ^2^Dipartimento di Medicina Interna Fondazione San Raffaele-G.Giglio, Cefalù (Palermo), Italy; ^3^Istituto di BioMedicina ed Immunologia Molecolare del CNR, Palermo, Italy

## Abstract

The distribution of the T29C TGF*β*1 gene polymorphism was analyzed in 198 hypertensives with left ventricular hypertrophy (LVH) and in 235 hypertensives without LVH. Circulating TGF*β*1 levels, procollagen type III levels, microalbuminuria, and left ventricular geometry and function were evaluated in all the hypertensives with LVH subgrouped according to T29C TGF*β*1 gene polymorphism. Circulating TGF*β*1 was evaluated by ELISA technique, procollagen type III by a specific radioimmunoassay, microalbuminuria by radioimmunoassay, and left ventricular geometry and function by echocardiography. All groups were comparable for gender, age, and sex. Regarding T29C TGF*β*1 gene polymorphism, prevalence of TC or CC genotypes was significantly (*P* < .05) higher in hypertensives with LVH than hypertensives without LVH TC and CC LVH hypertensives were characterized by a higher prevalence of subjects with microalbuminuria (*P* < .05 TC and CC versus TT), by increased levels of TGF*β*1, procollagen type III, urinary albumin excretion, LVM, LVM/h_2.7_, and lower values of left ventricular ejection fraction (*P* < .05 TC and CC versus TT). Our data suggest that T29C TGF*β*1 gene polymorphism was associated with clinical characteristics adequate to recognize a subset of LVH hypertensives with a higher severity of hypertension.

## 1. Introduction

Hypertension represents the most common powerful risk factor for cardiovascular morbidity and mortality [1]. High blood pressure is associated with adverse morphological and functional changes in the cardiovascular and renal system, including left ventricular hypertrophy (LVH), microalbuminuria and progressive renal and heart disease. 

 The disproportional accumulation of fibrous tissue is the major characteristic of the adverse structural remodelling of cardiac tissue in hypertensives promoting systolic and diastolic dysfunction [2–5].

Transforming growth factor *β*1 (TGF*β*1) is a multifunctional cytokine and its gene has been found in position 19q13.2, 19q13.1 on chromosome 19 and it has 7 exons and the length of the whole gene is 17.52 kb. 

TGF*β*1 overproduction acts on cardiomyocytes as well as on cardiac fibroblasts inducing cardiac fibrosis and hypertrophy [6, 7]. In addition, the reduction in circulating TGF*β*1 through a block of the renin-angiotensin system (RAS) was reported to be associated with an improvement of renal function and a reversion in LVH [8, 9]. Moreover, recent experimental data indicate that blockade of the TGF*β*, through a novel orally specific inhibitor of the TGF*β* receptor 1, results in significant improvement of deleterious cardiac remodelling after infarction [10]. 

Production of TGF*β*1 is in part under genetic control [11]. Eight single nucleotide polymorphism (SNP_s_) have been described in TGF*β*1 gene and related to its production and to hypertension and cardiovascular disease [11, 12]. Two SNP_s_ are located at positions 29 and 74 of the translated sequence of TGF*β*1 and give rise to amino acid substitutions at positions 10 (Leu^10^→Pro) and 25 (Arg^25^→  Pro) in the signal peptide of TGF*β*1, respectively [12, 13]. The T29C was reported to influence steady-state concentrations of TGF*β*1 mRNA in peripheral blood mononuclear cells and serum levels of TGF*β*1, and the G74C was found to be related to TGF*β*1 production in peripheral blood leukocytes [13, 14]. In addition, the Arg^25^ allele was associated with risk of hypertension in the normotensives [12, 15] and with myocardial infarction [12, 16] compared to the Pro^25^ allele. On the contrary, no studies have been addressed the evaluation of the role of T29 → C polymorphism of TGF*β*1 gene on left ventricular geometry and function in hypertensive patients [14, 16].

The aim of this study was to investigate the relationship between T29C TGF*β*1 gene polymorphism (rs1800470), LVH and clinical severity of hypertension. 

In particular, circulating TGF*β*1, procollagen type III levels, microalbuminuria, left ventricular geometry and function were evaluated in hypertensive patients and related to genotype profile.

## 2. Materials and Methods

### 2.1. Patients

Subjects eligible for the study were screened at the antihypertensive center of the Department of Internal Medicine, University of Palermo (Italy). The study population consisted of hypertensive subjects with age ≤65 years. Each patient gave a written consent after received a detailed description of study procedure. The study was approved by Ethics Committee of our Institution. Subjects under antihypertensive treatment or with a casual blood pressure (SBP) ≥140 mmHg and/or with casual diastolic blood pressure (DBP) ≥90 mmHg obtained with a standard sphygmomanometer after 5 minute of rest at three independent occasions with patients sitting were considered hypertensives. Exclusion criteria included secondary hypertension, endocrinal disease and diabetes mellitus, cardiovascular diseases (defined as myocardial infarction and recent stroke within previous 6 months, heart failure), severe chronic renal failure, alcoholism and psychiatric problems.

433 hypertensive subjects fulfilled the inclusion criteria and they were grouped according to the presence or absence of LVH, following standard echocardiographic criteria. In particular all the hypertensives with LVM/h^2.7^≥50 g/m^2.7^for men and ≥47 g/m^2.7^for women were considered to have LVH [19]. Accordingly, 198 hypertensives with LVH and 235 without LVH were recognized and studied. In addition 94.4% (187/198 pts) of LVH hypertensives and 94.9% (223/235 pts) of no-LVH hypertensives were under antihypertensive treatment at the beginning of the study. The percent of antihypertensive drugs utilized, such as duration of treatment, was not significantly different in all hypertensive groups (Table 1), also when subgrouped according to T/C genotypes (Table 2).

### 2.2. DNA Isolation and Genotyping

Genotyping was performed by investigators blinded to clinical status.

Peripheral venous blood was collected in EDTA from all the patients and stored at −70°C.

The PCR approach was used to analyze SNP in the coding regions of TGF*β*1. 

The study of the TGB*β*1 polymorphism was done by analyzing the sequence. The polymorphism is on exon 1 at +869 from the beginning of the transcription and generate an amino acid substitution in position 10 (Leu → Pro). This study analyzed the genotypes derived from this substitutions. 

 PCR was performed on purified DNA obtained using the GenElute Blood Genomic Dna Kit by Sigma: which provided sequence-specific oligonucleotide primers 

T869C: 

Forward 5′-TTCCCTCGAGGCCCTCCTA -3′

Reverse 5′-GCCGCAGCTTGGACAGGAT-3′

Briefly, PCR reactions were carried out in a total volume of 50-*μ*L containing approximately 5 *μ*L of genomic DNA(0.1 *μ*g/*μ*L), 2 *μ*L of forward and reverse primers (100 ng/*μ*L), 5 *μ*L of 10 × reaction buffer (160 mM (NH_4_)_2_SO_4_, 670 mM Tris-HCl (pH 8,8 at 25°C), 15 mM MgCl_2_, 0, 1% Tween 20), 4 *μ*L of 2 mM dNTPs (invitrogen), 4 *μ*L of DMSO, 0, 1u of Taq Polymerase.

Amplification was carried out in a Robocycler using cycle parameters of 3 minutes and 30 seconds at 95°C (initial denaturation), 35 cycles of 95°C for 45 seconds (denaturation), 62°C for 30 seconds (primer annealing) and a final extension for 10 minutes at 72°C.

T869C: the PCR generated amplicons with a fragment size of 294 Bp.

**Figure 1:**
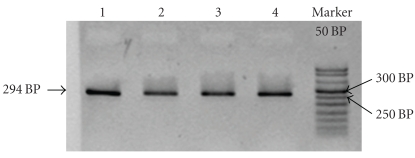
2% agar gel for confirming the amplification fragment (294 Bp). Subject 1–4, M marker 50 Bp (invitrogen).

All PCR products were resolved on 2% agarose gel with 3 *μ*L of ethidium bromide (Figure 1).

### 2.3. Sequencing

**Figure 2:**
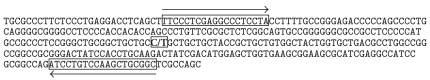
Localization scheme of the polymorphism at +869 C/T, situated inside exon 1, positions of the forward and reverse primers of the 294 Bp fragment are indicated.

PCR products were sequenced to genotype in all the subjects. PCR reactions in 50 *μ*L were directly sequenced by MWG (the full name is Eurofins MWG Operon, see http://www.eurofinsdna.com/home.html) and from sequence electropherograms was analysed the presence of single-nucleotide polymorphisms T869C in all the subjects (Figure 2). The distribution of SNP TGF*β*1 gene between both hypertensive groups were reported in Table 1.

### 2.4. Biochemical Measurements

Patients underwent a general analytical laboratory parameters profile including BUN, creatinine and clearance, glycaemia, electrolytes (serum sodium, potassium, chloride), cholesterol by routine laboratory methods.

Peripheral venous blood was obtained from each patient and the sera were isolated and stored at −70°C. TGF *β*1 levels were determined by using a solid-phase specific sandwich ELISA technique (R&D Systems, Inc. Minneapolis, USA) as previously described [17]. The interassay and intra-assay variations for determining TGF*β*1 were 8% and 6%, respectively. The sensitivity, hence minum level of detection of TGF*β*1 by sandwich ELISA, was 5 pg/mL. 

To determine amino-terminal PIIIP, blood samples were taken from each patient and stored at 40°C until manipulation. PIIIP were determined by using a specific radioimmunoassay (Orion Diagnostic Finland), as previously described [18]. The sensitivity of PIIIP was 1 ng/mL, the intra-assay variations ranged from 1.7% to 4.3% and interassay variations from 3.2% to 5.3%.

### 2.5. Urinary Albumin Excretion (UAE)

To eliminate the intra-individual day-to-day variability of UAE, three consecutive 24-hour urine collections were used. In addition, to assess the completeness of a 24 hours urine collection, measurements of urinary rate of clearance of creatinine were evaluated. UAE was measured by radioimmuno-assay (limit of detection, 0.1 mg/dL; Inter-assay coefficient 3.5%). Microalbuminuric patients were defined as a level of UAE ≥20 and <300 mg/24 hours.

### 2.6. Echocardiographic Measurements

All patients underwent an echocardiography examination M and B-mode, by a computerized echocardiography (ESAOTE, Italy) for the determination of following parameters: left ventricular telediastolic internal diameter (LVIDd), interventricular septum (IVSTd), and posterior wall thickness (PWTd). The Penn convention was used to calculate left ventricular mass (LVM). LVM was normalized for height to the 2.7 power [19]. The relative wall thickness (RWT) by formula [(PWTd/LVIDd) × 2] was also calculated. Ejection fraction from left ventricular end-diastolic and end-systolic volumes was measured from the apical four chamber view, using the ellipsoidal single-plane algorithm. Mean ejection fraction was automatically calculated by the echocardiographic processing system. In our laboratory the ejection fraction calculated over five consecutive beats permitted optimal reproducibility and accuracy [20]. 

LV relaxation and filling were evaluated by pulsed-wave Doppler interrogation of the LV inflow tract from the apical four-chamber view, with the sample volume placed at the tips of the mitral valve. After a stable signal of the transmitral flow velocity was obtained, the Doppler cursor was moved toward the LV outflow tract in the apical five-chamber view for recording both mitral and aortic signals, including the closing click of the aortic valve and the opening click of the mitral valve. Doppler signals were recorded at high speed (80–120 mm/s) with the subjects in held expiration. An average of five beats was used for analysis. 

Isovolumic relaxation time (IVRT) was calculated as the time from the closure click of the aortic valve to the opening click of the mitral valve. When either the closing or opening click was not identified, the time from the end of the aortic flow to the onset of mitral flow from the continuous wave interrogation of the LV inflow-outflow tract was used. Peak early transmitral flow velocity (*E*), peak late transmitral flow velocity (*A*), and the deceleration time of *E* velocity (DTE) were measured at the tips of mitral leaflets at the maximum amplitude of *E* velocity. DTE was measured as the time from peak *E* velocity to the time when *E* wave descent intercept the zero line.

### 2.7. Statistical Analysis

No sample size was calculated for the lack of any information about the main goal of the study.

Continuous variables are reported as mean ± SD. Absolute and relative frequencies are reported when appropriate. For continuous variables, comparisons among groups were performed by Kruskall-Wallis test as non parametric analysis of variance. Multiple pairwise comparisons were made by the Critchlow-Fligner method. Contingency tables were analyzed by the *Q* ^2^ test or the Fisher exact test when possible. Pairwise comparison between frequencies were made by *Z*-test after Chi-square statistical significant value. A two tailed *P* value <.05 was considered significant. 

Logistic regression analysis, according to Hosmer and Lemeshow method [21] has been used to investigate association between TT or TC plus CC genotypes and both laboratory and clinical measurements. Continuous variables were put into the model as quintiles.

## 3. Results

The results of the study are presented in Tables 1–5.

### 3.1. Distribution of T29C TGF*β*1 Genotypes in Hypertensives With and Without LVH

The distribution of T29C genotypes in both hypertensive groups has been reported in Table 1. The prevalence of TC (*P* = .0434), and TC plus CC (*P* = .0466) genotypes, was significantly higher in hypertensives with LVH than hypertensives without LVH (64.7% versus 49.8%, and 84.9% versus 61.4%, respectively). 

Genotype frequency distribution in the two groups of hypertensives occurred according to Hardy-Weinberg proportions.

Since genotype frequency distribution was not significant different in hypertensives without LVH, only hypertensives with LVH were further subdivided into three groups, according to T/C genotypes.

### 3.2. Distribution of Clinical Characteristics in Hypertensives With LVH Subgrouped According to T29C TGF*β*1 Genotypes

The association between genotypes and clinical characteristics in the three groups of hypertensives with LVH have been reported in Table 2. All the groups were homogeneous regarding to age, BMI, WHR and blood pressure. LVH hypertensives with TC or CC genotype were characterized by a significant higher prevalence of subjects with microalbuminuria (*P* < .05 TC and CC versus TT).

No significant difference in the prevalence and duration of antihypertensive drugs utilized was observed among the groups (Tables 1 and 2). In addition no significant difference also in statin administration was observed among the groups.

### 3.3. Circulating TGF*β*1, Type III Collagen, Urinary Albumin Excretion and Echocardiographic Parameters

Urinary albumin excretion, circulating TGF*β*1, PIIIP, LVM and LVM/h^2.7^levels were significantly (*P* < .05) higher and left ventricular ejection fraction values were significantly (*P* < .05) lower in LVH hypertensives with TC or CC genotype than those detectable in LVH hypertensives homozygous for allele T (Tables 3 and 4).

## 4. Logistic Regression Analysis

This analysis indicated an association between higher levels of PIIIP and TC or CC genotypes, even if adjusted for LVM/h^2.7^ and urinary albumin excretion values (Table 5).

## 5. Discussion and Conclusion

To our knowledge this is the first study to investigate the impact of TGF*β*1 Leu → Pro at codon 10 polymorphism (rs1800470) on left ventricular geometry and function in hypertensive patients. Our data indicate a higher prevalence of TC and CC Leu^10^→Pro polymorphism in hypertensives with LVH than hypertensives without LVH, associated to some unfavorable clinical characteristics of hypertension. In fact, LVH hypertensive subjects with TC or CC genotype were characterized by a higher prevalence of subjects with microalbuminuria, higher value of LVM and lower left ventricular ejection fraction. In our opinion, this association doesn't reflect unknown differences in population ancestry between the two hypertensive groups. We consider the probability of false positive inference attributable to population studied rather small because the two hypertensive groups were recruited from an ethnically homogenous population. In this context recent results from Xu et al. [22] revealed a genetic association of TGF*β*1 + 915 Arg → Pro at codon 25 polymorphism with LVH in a Chinese hypertensive population, while the codon 10 genotypes did not show any association to LVH. Even if in this study we have analyzed only TGF*β*1 Leu → Pro at codon 10 polymorphism, our recent unpublished data indicated no association between TGF*β*1 + 915 Arg → Pro at codon 25 polymorphism and hypertension in a Caucasian hypertensive population. These contrasting data may be explained by ethnicity of two hypertensive populations considered (Caucasian and Chinese).

Although changes in the heart caused by hypertension are well known, the effective mechanisms are not entirely clarified. Despite the role of hemodynamic effects and growth factors have been largely reported, other metabolic and inflammatory factors have to be also considered [23].

Literature data and the results of our previous studies indicate an overproduction of circulating TGF*β*1 in hypertensives. Moreover, hypertensives with target organ damage (TOD) have higher circulating levels of TGF*β*1 than hypertensives without TOD. This finding seems to attribute an important role to TGF*β*1 overproduction in the pathophysiology of essential hypertension and its sequelae [24, 25]. The TGF*β*1 overproduction in hypertension may be explained by the effects of various factors, such as elevated angiotensin II, increased fluid shear stress and a differential expression of TGF*β*1 linked to DNA polymorphisms in the promoter [11]. Plasma concentrations of active and also of acid-activable TGF*β*1 is predominantly under genetic control (heritability estimate 0.54). Up regulation of TGF*β*1 system in monocytes of hypertensive patients and association of TGF*β*1 gene polymorphism with risk of hypertension suggests that quantitative difference in TGF*β*1 production may determine the intensity of the process of vascular remodelling and therefore influence overall susceptibility to the development of hypertension [26]. On the other hand experimental data indicate that abnormalities in responsiveness to TGF*β*1 overproduction, as evidenced by collagen formation, may represent a pathophysiological molecular mechanism in hypertension [27, 28]. The results from ECTIM study [12] suggest that TGF*β*1 Arg25-Pro polymorphism might be associated with hypertension but does not address the issue of quantitative phenotypes related to TGF*β*1 production in relation to hypertension. On the contrary no study has been addressed the evaluation of the role of Leu-to-Pro polymorphism at codon 10 to explain whether the substitution has functional and biological importance, or it could affect protein transport. In view of this we have now shown that the serum concentration of TGF*β*1 was significantly different in LVH hypertensives subgrouped according to T29 → C polymorphism. It is possible that the T29 → C polymorphism of the TGF*β*1 gene is linked to some other genes that are actually responsible for the development of hypertension.

Moreover, the significant change in circulating TGF*β*1 values among LVH hypertensive groups indicated that TC or CC genotypes could be able to induce quantitative change in the production of the cytokine and it might affect also the function of signal peptide, perhaps influencing intracellular trafficking or export efficiency of preprotein [15].

In addition, our data suggest some clinical considerations. First, LVH hypertensive subjects with TC or CC genotypes were characterized by a higher value of microalbuminuria, LVM and by a systolic left ventricular dysfunction. This might indicate that hypertension in these subjects has to be considered more severe. Another aspect of our study that seems to be interesting is related to the higher collagen production in LVH hypertensive subjects with TC or CC genotype than in those homozygous for allele T. This finding is further supported by analysis of logistic regression indicating that PIIIP may be considered the most important marker associated to T and C alleles. In fact, analysis of odd ratio indicates that the risk of association with T or C allele increases eight fold for each PIIIP quintile variation. In our opinion this finding might further support our previous results [5, 23] indicating that overproduction in circulating TGF*β*1 may contribute to the progression of renal and cardiovascular damage in obese and/or hypertensive subjects. In particular, the present study emphasizes the unfavourable effects of TGF*β*1 overproduction on left ventricular geometry and function that might be mediated by an higher collagen production [5, 24, 29]. In view of this the association of TC or CC with a higher clinical severity of hypertension seems to indicate that TGF*β*1 is a susceptibility locus for hypertension. A potential limit of this study was to have computed no a priori evaluation of the *β* error and consequently the power of our statistical analysis. However this involves considerations only about the sample size. Accordingly the negative results of our study has needed further evaluations.

In conclusion our data are attractive to indicate that LVH hypertensive subjects with TC or CC genotypes (Leu^10^  → Pro polymorphism, rs 1800470) might be considered a particular subset of LVH hypertensives with a more severity of hypertension. However, the present results require verifications in other populations, since it is well known that hypertension is under the control of many genes that contribute modest individual effects, and TGF*β*1 may act in concert with other hypertension susceptibility loci.

## Figures and Tables

**Table 1:** Clinical characteristics and distribution of Leu^10^→Pro^10^ genotypes in hypertensives (Ht) with and without left ventricular hypertrophy (LVH).

	Ht with LVH	Ht without LVH	*P*=
*n.*	198	235	
Age	50 ± 10	49 ± 10	.300
Gender (F/M)	90/108	124/111	.590
BMI (Kg/m^2^)	25 ± 6	24 ± 6	.085
WHR	0.86 ± 0.1	0.85 ± 0.1	.300
SBP (mm/Hg)	144 ± 15	143 ± 16	.505
DBP (mm/Hg)	88 ± 9	87 ± 10	.279
MBP (mm/Hg)	106 ± 9	105 ± 9	.250
Ht treated (%)	94.4% (187/198)	94.9% (223/235)	.987*
Diuretics	9% (17/187)	12.1% (27/223)	.394*
ACEi	58.8% (110/187)	61.8% (138/223)	.605*
ARB	42.8% (80/187)	40.3% (90/223)	.607*
CaCB	11.8% (22/187)	17.3% (30/223)	.199*
Duration of treatment (yrs)	6.5 ± 3.3	6.2 ± 3.9	.393
T29C (Leu^10^→Pro)			
TT	15.1% (30/198)	30.6% (72/235)	.0418
TC	64.7%* (128/198)	49.8% (117/235)	.0434
CC	20.2%* (40/198)	19.6% (46/235)	.0472
TC + CC	84.9%	69.4%	.0466

*z test.

F: Female; M: Male; BMI: Body mass index; WHR: Waist hip ratio; SBP: systolic blood pressure; DBP: diastolic blood pressure;

MBP: mean blood pressure. ACEi: angiotensin converting enzyme inhibitors; ARB: Angiotensin receptor blockers; CaCB: calcium channel blockers. n.s. = not significant.

**Table 2:** Distribution of clinical measurements in the hypertensives with left ventricular hypertrophy subgrouped according to Leu^10^→  Pro^10^ TGF*β*1 genotypes*.

	TT *n.* 30	TC *n.* 128	CC *n.* 40
Gender(F/M)	16/14	60/68	14/26
Age	55 ± 10	50 ± 9	50 ± 9
Weight (Kg)	69 ± 11	72 ± 10	70 ± 9
Height (m)	1.67 ± 0.1	1.65 ± 0.1	1.68 ± 0.1
BMI (Kg/m^2^)	24 ± 2	25 ± 2	24 ± 3
WHR	0.85 ± 0.1	0.87 ± 0.1	0.87± 0.1
SBP (mmHg)	143 ± 16	146 ± 16	141 ± 16
DBP (mmHg)	87 ± 10	90 ± 11	89 ± 8
MBP (mmHg)	105 ± 11	108 ± 11	106 ± 9
Antihypertensive drugs			
Diuretics	3/30 (10%)	10/128 (7.9%)	4/40 (10%)
ACEi	16/30 (53.4%)	75/128 (58.6%)	19/40 (47.5%)
ARB	12/30 (40%)	58/128 (42.1%)	14/40 (35%)
CaCB	4/30 (13.3%)	12/128 (8.4%)	6/40 (15%)

F: Female; M: Male; BMI: Body mass index; WHR: waist hip ratio; SBP: Systolic blood pressure; DBP: Diastolic blood pressure; MBP: Mean blood pressure; ACEi: angiotensin converting enzyme inhibitors; ARB: Angiotensin receptor blockers; CaCB: calcium channel blockers.

*All variables were not significantly different among the groups.

**Table 3:** Renal measurements, Circulating TGF*β*1 and Pro-collagen III in hypertensives with left ventricular hypertrophy subgrouped according to Leu^10^→  Pro^10^ TGF*β*1 genotypes.

	TT *n.* 30	TC *n.* 128	CC *n.* 40
BUN (mg/dL)	39 ± 6	38 ± 7	39 ± 8
Creatinine (*μ*mol/L)	61.9 ±17.7	79.6 ± 17.7	79.6 ± 17.7
Clearance (mL/min)	108 ± 30	103 ± 38	102 ± 32
UAE (mg/24h)	51 ± 40	113 ± 36*	92 ± 69*
Microalbuminuric	5/30	80/128	21/40
pts (%)	(16.7%)	(62.5%)*	(52.5%)*
TGF*β*1 (ng/mL)	45 ± 22	60 ± 15*	58 ± 11*
PIIIP (U/L)	0.60 ± 0.1	0.71 ± 0.1*	0.65 ± 0.1*

BUN: Blood urea nitrogen; TGF*β*1: Transforming growth factor *β*1; PIIIP: amino-terminal propeptide of type III procollagen; UAE: Urinary albumin excretion; pts: patients.

*P < .05 versus TT.

**Table 4:** Left ventricular geometry and function in hypertensives with left ventricular hypertrophy subgrouped according to Leu^10^→  Pro^10^ TGF*β*1 genotypes.

	TT *n.* 30	TC *n.* 128	CC *n.* 40
LVIDd (mm)	49 ± 3	48 ± 4	48 ± 5
IVSTd (mm)	11 ± 1	11.9 ± 2	11,8 ± 2
PWTd (mm)	9.6 ± 1	10.5 ± 2	10.6 ± 1
RWT	0.39 ± 0.1	0.44 ± 0.1	0.44 ± 0.1
LVM (gr)	197 ± 33	228 ± 40*	216 ± 55*
LVM/h^2.7^ (gr/h^2.7^)	53 ± 8	59 ± 10*	56 ± 9*
EF %	63 ± 3	57 ± 2*	56 ± 3*
E/A	1.5 ± 0.4	1.4 ± 0.3	1.4 ± 0.4
DTE (ms)	202 ± 38	204 ± 42	205 ± 44
IVRT (ms)	80 ± 12	78 ± 15	81 ± 14

LVIDd: Left ventricular telediastolic internal diameter; IVSTd: Interventricular septum thickness; PWTd: Posterior wall thickness; LVM: Left ventricular mass; LVM/h^2.7^: Left ventricular mass indexed 2.7; RWT: Relative wall thickness; EF: Ejection fraction; E/A: Peak early transmitral flow/peak late transmitral flow; DTE:* E *deceleration time; IVRT: Isovolumic relaxation time.

*P < .05 versus TT.

**Table 5:** *Logistic regression analysis. *Deviance (likelihood ratio) chi-square = 14,281753 df = 3 *P* = .0025.

Independent variable	O.R*	*P*=
PIIIP	8.1 (C.I: 1.50–15.20)	.0466
UAE	4.3 (C.I: 0.93–8.43)	.0814
LVM/h^2.7^	2.58 (C.I: 0.57–4.34)	.203

logit *y* = −0,142938 + 2,090717 PIIIP +1,461049 UAE + 0,949264 LVM/h^2.7^.

logit *y* = 1 for TC + CC genotypes.

logit *y* = 0 for TT genotype.

O.R.= odd ratio; C.I = Confidence interval.

*Odd ratio is referred to quintile variation of each variable.
